# Molecular Features Behind Formation of α or β Co-Crystalline and Nanoporous-Crystalline Phases of PPO

**DOI:** 10.3389/fchem.2021.809850

**Published:** 2022-01-25

**Authors:** Manohar Golla, Antonietta Cozzolino, Baku Nagendra, Emanuele Vignola, Christophe Daniel, Paola Rizzo, Gaetano Guerra, Finizia Auriemma, Massimo Christian D’Alterio

**Affiliations:** ^1^ Dipartimento di Chimica e Biologia and INSTM Research Unit, Università Degli Studi di Salerno, Fisciano, Italy; ^2^ Dipartimento di Scienze Chimiche, Università di Napoli “Federico II”, Napoli, Italy

**Keywords:** guest molecular volume, guest solubility in water, solubility parameters, DFT calculations, dispersive energy calculations

## Abstract

Guest molecular features determining the formation of α and β phases of poly(2-6-dimethyl-1,4-phenylene) oxide (PPO) are explored by collecting literature data and adding many new film preparations, both by solution casting and by guest sorption in amorphous films. Independently of the considered preparation method, the α-form is favored by the hydrophobic and bulky guest molecules, while the hydrophilic and small guest molecules favor the β-form. Furthermore, molecular modeling studies indicate that the β-form inducer guests establish stronger dispersive interactions with the PPO units than the α-form inducer guests. Thus, the achievement of co-crystalline (and derived nanoporous crystalline) α- and β-forms would result from differences in energy gain due to the host–guest interactions established at the local scale.

## 1 Introduction

An engineering thermoplastic polymer, poly(2-6-dimethyl-1,4-phenylene) oxide (PPO), is always amorphous by melt processing (Toi et al., 1982; Guerra et al., 1991; Tsujita, 2003; Yang and Knauss, 2015; Minelli et al., 2017), while it can be crystallized by solution processing as by sorption of guest molecules in amorphous samples (Alentiev et al., 1998; Khulbe et al., 2000; Khayet et al., 2004; Sterescu et al., 2007; Daniel et al., 2011; Nagendra et al., 2019; Alentiev et al., 2021).

In particular, two completely different classes of co-crystalline (CC) forms with low-molecular-mass guest molecules can be obtained. The CC forms of Class i are formed only with a few specific guests (α-pinene, tetralin, and decalin), exhibit regular polymer helices, and give rise to highly ordered crystalline phases (Barrales-Rienda and Fatou, 1971; Horikiri, 1972; Hurek and Turska, 1984; Tarallo et al., 2012). The CC forms of Class ii are instead formed with many guests and exhibit a different chain conformations and less ordered crystalline phases (Daniel et al., 2011; Nagendra et al., 2019; Golla et al., 2020a; Alentiev et al., 2021).

The CC forms of Class ii can be divided into two subclasses (α and β) with different values of the chain periodicities (*c* = 0.528 nm and *c* = 0.547 nm) and different polymer packing (with main equatorial reflections at 2θ_CuKα_ ≈ 4.5°, 7.1°, 11.2°, 14.9°, and 5.2°, 7.7°, 12.8°) (Daniel et al., 2011; Nagendra et al., 2019).

Both the CC α- and β-forms can be obtained by guest-induced crystallization of amorphous samples or solution processing. However, in previous studies, we have found that most guest molecules give only CC α-form or CC β-form, independently of the crystallization procedure, and, as a consequence, have been named α or β guests of PPO.

After guest removal, the CC phases of Class i become amorphous (Barrales-Rienda and Fatou, 1971; Horikiri, 1972; Hurek and Turska, 1984; Tarallo et al., 2012), while the CC phases of Class ii can produce two different nanoporous crystalline (NC) forms, i.e., crystalline forms with a density lower than that of the corresponding amorphous phase. These NC forms were also named α and β because they maintain wide-angle x-ray diffraction (WAXD) patterns similar to those of the corresponding CC forms (Nagendra et al., 2019). The NC α- and β-forms exhibit crystalline densities of nearly 0.93 and 0.95 g/cm^3^, respectively, being much lower than that of the corresponding amorphous phase (1.04 g/cm^3^) (Daniel et al., 2011; Nagendra et al., 2019).

A study on the stability of the NC α- and β-forms to the uptake of β and α guests, respectively, has shown that the NC α phases by sorption of a β guest lead to CC α phases, while on the contrary, the NC β phases by sorption of an α guest can lead to CC α phases (Nagendra et al., 2020). This clearly indicates a higher thermodynamic stability of the CC α phases (Nagendra et al., 2020). A recent study has also shown that NC α phases have generally higher melting temperatures with respect to NC β phases (Nagendra et al., 2021a).

Along with another commercial thermoplastic polymer (i.e., syndiotactic polystyrene) (De Rosa et al., 1997; Petraccone et al., 2008; Gowd et al., 2009; Acocella et al., 2015; Itagaki et al., 2017), PPO is the only other polymer known to give NC phases. NC polymers (mainly as films, fibers, and aerogels) (Daniel et al., 2013a; Wang and Jana, 2013; Daniel et al., 2016; Krishnan et al., 2021) can be helpful in many applications as molecular separation membranes (Daniel et al., 2011; Galizia et al., 2012; Daniel et al., 2013b; Galizia et al., 2013) for the purification of air (Daniel et al., 2021) and water (Cozzolino et al., 2021) from organic compounds, molecular sensors (Pilla et al., 2009; Lova et al., 2016), and catalysts (Vaiano et al., 2014; Litta et al., 2021). Moreover, the CC phases with active guest molecules can be useful for many different kinds of applications (Guerra et al., 2012), mainly as antimicrobial (Albunia et al., 2014; Rizzo et al., 2019a; Golla et al., 2021).

The present study collects literature data as well as new data relative to the influence of the chemical nature of the guest on the formation of CC and NC α or β phases of PPO. PPO crystallization is conducted by two different routes: solution casting and guest-induced crystallization of amorphous films. The aim is to try to establish, with the support of molecular modeling, guest molecular features determining the formation of α or β crystalline phases.

## 2 Experimental Section and Characterization Techniques

An ultrahigh molecular weight (M_w_ = 350 kg/mol, P6130 grade) of PPO was kindly supplied by SABIC, Milan. The following compounds: acetonitrile, *p*-xylene, 1,3-dichlorobenzene, perchloroethylene, *m*-xylene, ethylbenzene, 1,2-dichloropropane, 1,4-dioxane, ethyl acetate, diethyl ether, and tetrahydrofuran were purchased from Aldrich and used without further purification.

Amorphous PPO films were obtained by casting at 60°C of a 1.5 wt% chloroform solution. CC PPO films were prepared by immersion of amorphous PPO films in the liquid guest at room temperature, and this method is indicated in Table 1 as guest-induced crystallization (GIC). Most CC PPO films were also obtained by casting of 1.5 wt% polymer solutions, and this method is indicated in Table 1 as casting. NC films were obtained by room temperature sorption/desorption treatment of CC films with acetonitrile, which is a very volatile guest of co-crystalline phases of PPO. Film thickness is generally in the range of 40–70 µm.

**TABLE 1:** Solubility parameter of the guest molecules vs the corresponding crystalline phases of PPO. (For the sake of comparison, the solubility parameter of PPO is also included.)

Guest molecules	Solubility parameter (MPa^1/2^)	References for crystal form	Preparation technique	Crystal form
Diethyl ether	15.64	Present work	GIC	β-form
TCA	17.45	Nagendra et al. (2019), Nagendra et al. (2021b)	casting and GIC	α-form
*m*-Xylene	17.7	Present work	casting and GIC	Mixed
*p*-Xylene	17.7	Present work	casting and GIC	α-form
CCl_4_	17.81	Nagendra et al. (2019), Nagendra et al. (2021b)	casting and GIC	α-form
Limonene	17.82	Nagendra et al. (2019), Nagendra et al. (2021a)	casting and GIC	α-form
Ethylbenzene	17.86	Present work	casting and GIC	β-form
Mesitylene	18.01	Golla et al. (2020b), Nagendra et al. (2021b)	casting and GIC	α-form
*o*-Xylene	18.09	Nagendra et al. (2019), Nagendra et al. (2021b)	casting and GIC	α-form
Ethyl acetate	18.15	Present work	GIC	β-form
Toluene	18.16	Nagendra et al. (2019), Nagendra et al. (2021b)	casting and GIC	α-form
Benzene	18.5	Nagendra et al. (2019)	casting	β-form
PPO	18.6	Khayet et al. (2004)	—	—
Hexanal	18.73	Nagendra et al. (2019)	GIC	β-form
1,2-Dichloropropane	18.92	Present work	casting and GIC	β-form
CHCl_3_	18.94	Nagendra et al. (2019)	casting	β-form
Trichloroethylene	19.01	Nagendra et al. (2019)	casting	α-form
Methyl ethyl ketone	19.05	Nagendra et al. (2019)	GIC	β-form
Dibenzyl ether	19.13	Nagendra et al. (2019), Nagendra et al. (2021b)	casting and GIC	α-form
Methyl benzoate	19.45	Nagendra et al. (2019), Nagendra et al. (2021b)	casting and GIC	β-form
Tetrahydrofuran	19.46	Present work	GIC	β-form
Chlorobenzene	19.58	Nagendra et al. (2019); Nagendra et al. (2021a)	casting and GIC	α-form
DCA	19.88	Nagendra et al. (2021a)	casting and GIC	β-form
Carvone	19.9	Nagendra et al. (2021a), Nagendra et al. (2021b)	casting and GIC	α-form
Eugenol	20.03	Present work and Nagendra et al. (2021b)	casting& GIC	α-form
Perchloroethylene	20.28	Present work	casting and GIC	β-form
1,4-Dioxane	20.46	Present work	casting and GIC	β-form
1,2-Dichlorobenzene	20.47	Nagendra et al. (2019), Nagendra et al. (2021b)	casting and GIC	α-form
Carbon disulfide	20.5	Toi et al. (1982)	casting	β-form
1,3-Dichlorobenzene	20.52	Present work	casting and GIC	α-form
Carvacrol	20.7	Golla et al. (2021)	GIC	α-form
1,2,4-Trichlorobenzene	21.31	Nagendra et al. (2019), Nagendra et al. (2021b)	casting and GIC	α-form

GIC, guest-induced crystallization on amorphous film.

X-ray diffraction patterns were obtained by a Bruker D2 automatic diffractometer with nickel-filtered CuKα radiation, operated at a step size of 0.03° with 164 s/step.

Fourier transform infrared spectroscopy (FTIR) was conducted with a Vertex 70 Bruker spectrophotometer. It is equipped with a deuterated triglycine sulfate detector and a Ge/KBr beam splitter, which are operated at 2.0 cm^−1^ resolution. A total of 32 scans were averaged to reduce the spectral noise.

The physical parameters of the guest molecules, such as solubility parameter (*δ*_*sp*_) (Hansen, 2007) and solubility in water at room temperature (Horvath, 1982; Yalkowsky and Dannenfelser, 1992; Yalkowsky et al., 2010; Reichardt and Welton, 2011; IUPAC-NIST Solubility Database, 2012), were collected from the literature.

The guest molecular volume was calculated from the following equation (Nagendra et al., 2021b):

$$V_{guest}=\frac{M}{\rho N_{A}},$$ (1)

where M and ρ are the molecular mass and density of the guest molecules, respectively. N_A_ is the Avogadro’s number (6.02 × 10^23^ molecules/mol).

We carried out the density functional theory static calculations with the Gaussian 09, Revision E.01 set of programs (Frisch et al., 2016). We adopted the B3LYP functional (Perdew, 1986; Becke, 1988) opportunely corrected with the dispersion term, keyword Empirical Dispersion = GD3BJ in the used package (Grimme, 2004; Grimme et al., 2004). All the atoms (C, H, O, and Cl) were electronically described with the standard split-valence basis set with a polarization function of Ahlrichs and co-workers (Schäfer et al., 1992), keyword SVP in Gaussian 09 E.01.

## 3 Results and Discussion

### 3.1 Guests Leading to α and β Crystalline Forms

**FIGURE 1:**
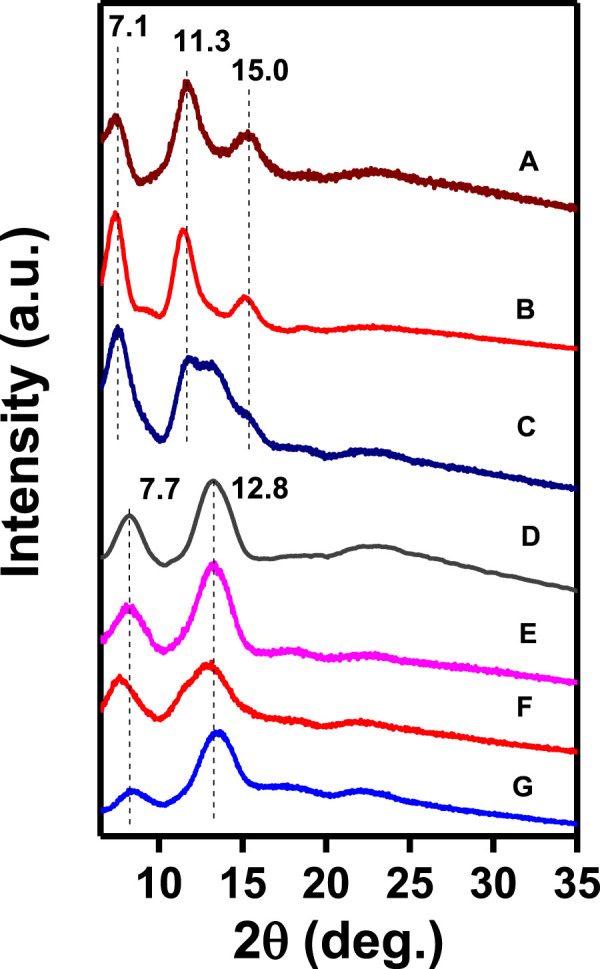
WAXD patterns of NC PPO films as obtained by solution casting, followed by guest extraction by acetonitrile: A) *p*-xylene, B) 1,3-dichlorobenzene, C) *m*-xylene, D) perchloroethylene, E) ethylbenzene, F) 1,2-dichloropropane, and G) 1,4-dioxane.

WAXD patterns of the NC PPO films (with thickness in the range 40–70 µm), as obtained by casting from 1.5 wt% polymer solutions in many different solvents, followed by guest removal by immersion in acetonitrile for 2 hours, are shown in Figure 1. The patterns of Figure 1 clearly show that the crystalline phases obtained from the polymer solutions in *p*-xylene (A) and 1,3-dichlorobenzene (B) exhibit *hk*0 diffraction peaks at 2θ_CuKα_ ≈ 7.1°, 11.3°, 15.0° typical of the α-form, while the crystalline phases obtained with perchloroethylene (D), ethylbenzene (E), 1,2-dichloropropane (F), and 1,4-dioxane (G) exhibit the *hk*0 diffraction peaks at 2θ_CuKα_ ≈ 7.7°, 12.8° typical of the β-form. The WAXD pattern of the film obtained by casting from *m*-xylene solution (C) shows diffraction peaks of both α and β crystalline forms.

**FIGURE 2:**
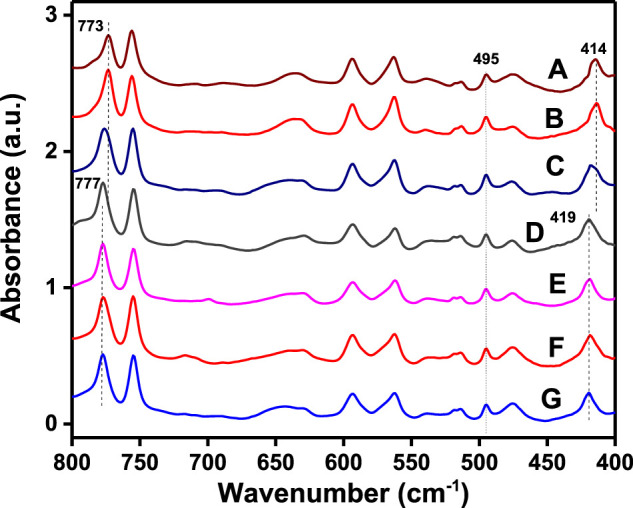
FTIR spectra of PPO films as obtained by solution casting, followed by guest extraction by acetonitrile: A) *p*-xylene, B) 1,3-dichlorobenzene, C) *m*-xylene, D) perchloroethylene, E) ethylbenzene, F) 1,2-dichloropropane, and G) 1,4-dioxane.

This information is confirmed by FTIR spectra of the same films of Figure 1, which are reported in Figure 2, for the spectral range 800–400 cm^−1^. In fact, Figures 2A,B show absorbance peaks typical of the α-form (at 773 and 414 cm^−1^), while the spectra of Figures 2D–G show absorbance peaks typical of the β-form (at 777 and 419 cm^−1^).

The crystalline forms (α or β) of films as crystallized by sorption of the same solvents in amorphous PPO films were also established by WAXD and FTIR measurements, like those of Figures 1, 2, respectively.

Table 1 collects in its last column the crystalline forms (α or β) of all films characterized in this study together with those already reported in the literature (Khulbe et al., 2000; Daniel et al., 2011; Nagendra et al., 2019; Golla et al., 2020b; Nagendra et al., 2021a; Nagendra et al., 2021b). In Table 1, the guest molecules are ordered on the basis of their solubility parameter (second column) while the used crystallization methods are indicated in the fourth column.

This fourth column of Table 1 clearly shows that the structure of the obtained crystalline forms does not depend on the crystallization method but only on the chemical nature of the guest used for polymer co-crystallization. Moreover, Table 1 shows the absence of any correlation between the structure of the CC forms (α or β) and the solubility parameter of the guest, which is generally expected to be relevant for host–guest co-crystallizations.

Based on the crystallization data of Table 1, we have explored possible correlations between the structural and physical properties of the considered guests and achievement of the α- and β-forms. We found the best correlations with molecular volume and solubility in water, as shown in Tables 2, 3, respectively.

**TABLE 2:** Guest molecular volume vs corresponding crystalline forms.

Guest molecules	Guest molecular volume (Ǻ^3^)	Crystal form
Dibenzyl ether	315.8	α-form
Limonene	269.0	α-form
Carvone	259.9	α-form
Eugenol	257.3	α-form
Carvacrol	255.3	α-form
Mesitylene	231.0	α-form
Methyl benzoate	208.6	β-form
1,2,4-Trichlorobenzene	206.4	α-form
*m*-Xylene	205.0	Mixed
*p*-Xylene	204.8	α-form
Hexanal	204.1	β-form
Ethylbenzene	203.5	β-form
*o*-Xylene	200.4	α-form
1,3-Dichlorobenzene	189.6	α-form
1,2-Dichlorobenzene	187.8	α-form
Toluene	175.9	α-form
Diethyl ether	172.6	β-form
Perchloroethylene	169.8	β-form
Chlorobenzene	168.4	α-form
TCA	167.8	α-form
1,2-Dichloropropane	162.3	β-form
Ethyl acetate	162.2	β-form
CCl_4_	160.7	α-form
Trichloroethylene	149.4	α-form
Methyl ethyl ketone	148.7	β-form
Benzene	148.1	β-form
1,4-Dioxane	141.6	β-form
Tetrahydrofuran	135.0	β-form
CHCl_3_	133.1	β-form
DCA	131.5	β-form
Carbon disulfide	99.8	β-form

**TABLE 3:** Guest solubility in 100 ml of water vs corresponding crystalline forms.

Guest molecules	Guest solubility in 100 ml of water at 25°C (mmol)	Crystal form
Limonene	0.010	α-form
Dibenzyl ether	0.020	α-form
1,2,4-Trichlorobenzene	0.027	α-form
Mesitylene	0.040	α-form
1,3-Dichlorobenzene	0.084	α-form
1,2-Dichlorobenzene	0.106	α-form
Perchloroethylene	0.124	β-form
*o*-Xylene	0.152	α-form
*m*-Xylene	0.150	Mixed
*p*-Xylene	0.154	α-form
Ethylbenzene	0.159	β-form
Chlorobenzene	0.439	α-form
CCl_4_	0.526	α-form
Toluene	0.564	α-form
Carvacrol	0.832	α-form
Carvone	0.865	α-form
TCA	0.967	α-form
Trichloroethylene	0.974	α-form
Eugenol	1.498	α-form
Methyl benzoate	1.491	β-form
Benzene	2.304	β-form
1,2-Dichloropropane	2.478	β-form
Carbon disulfide	2.836	β-form
Hexanal	4.992	β-form
CHCl_3_	6.701	β-form
DCA	8.700	β-form
Diethyl ether	81.62	β-form
Methyl ethyl ketone	323.1	β-form
Ethyl acetate	92.1	β-form
Tetrahydrofuran	686	β-form
1,4-Dioxane	1,140	β-form

In detail, Table 2 shows that all the considered guests with molecular volumes higher than 230 Å^3^ and lower than 149 Å^3^ lead to the α- and β-forms, respectively. As for the guest molecular volume, it is worth adding that it also has a key role in determining the orientation (with crystalline chain axis being preferentially parallel or perpendicular to the film plane) (Rizzo et al., 2019b) of PPO films (Nagendra et al., 2021b). Table 3 shows that all guests whose solubility is lower than 0.11 mmol per 100 ml of water and higher than 2 mmol per 100 ml of water lead to the α- and β-forms, respectively.

In summary, our data in Tables 1–3 show that, independently of the crystallization method, the α-form is favored by hydrophobic and bulky guest molecules while the hydrophilic and small guest molecules favor the β-form. Hence, more hydrophilic and smaller guest molecules favor the formation of the CC and NC crystalline β-forms, which are characterized by a higher chain periodicity (Nagendra et al., 2019).

The molecular modeling of the following section is aimed to rationalize the presently observed behavior.

### 3.2 Energy Evaluations Associated With the Interactions of Guest Molecules With PPO Units

**FIGURE 3:**
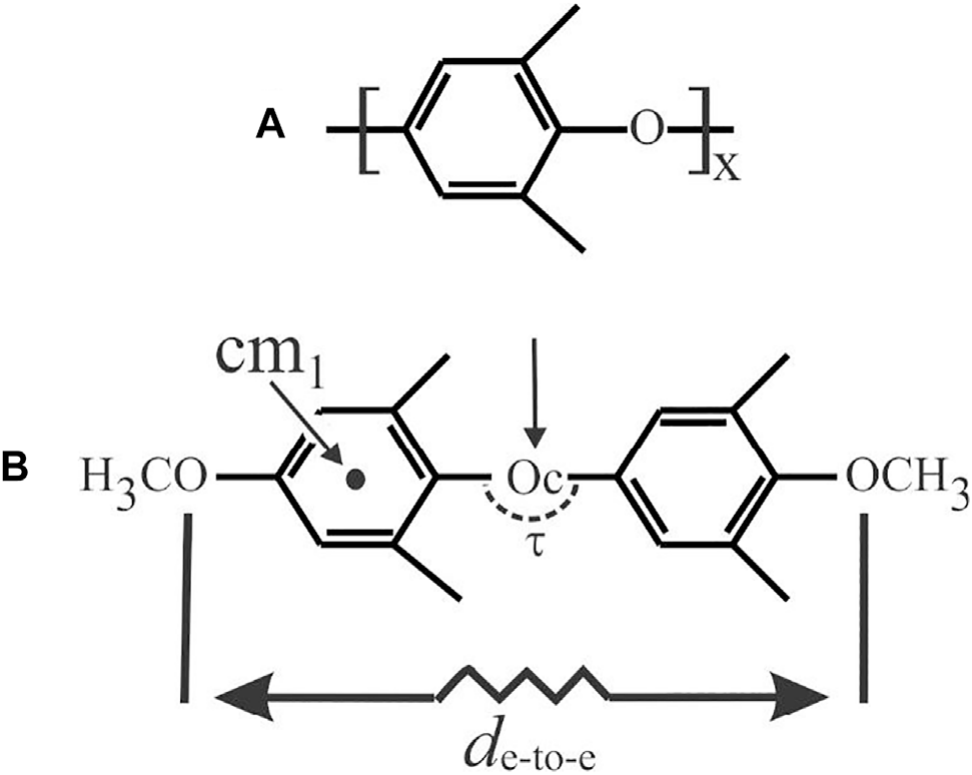
PPO polymer **(A)** and methyl-terminated model dimer **(B)** used in the calculations of potential energy. The center of mass of the phenyl ring cm_1_ and the model dimer Oc is indicated in **(B)** with an arrow. The valence angle at the central Oc atom Cph-Oc-Cph (τ) and the end-to-end distance *d*_e-to-e_ are indicated.

Potential energy calculations were performed in the hypothesis that the formation of the α and β CC and NC forms is driven by the host–guest dispersive interactions exerted at the local scale between a PPO monomeric unit and a guest molecule. In particular, the potential energy was calculated on the methyl-terminated model dimer of PPO as shown in Figure 3B, in the absence and presence of four kinds of probe molecules, namely 1,2-dichloroethane (DCA) and CHCl_3_, as examples of β-form inducer guests, and 1,1,1-trichloroethane (TCA) and CCl_4_, as examples of α-form inducer guests. For the sake of simplicity, the conformation of the model dimer was assumed in the extended state as in the repetition unit of the model chain of Supplementary Figure S2C of Nagendra et al. (2019). In the calculation, the model dimer was gradually stretched through deformation of the valence angle at the central Oc atom Cph-Oc-Cph (τ) (C and ph standing for central oxygen atom and carbon phenyl rings, respectively) over a range of values between 119 and 140° (Naumov and Ziatdinova, 1984; Baukova et al., 1994a; Baukova et al., 1994b; Nevalainen and Rissanen, 1994). The stretching state of the model dimer is measured by the increase of the end-to-end distance *d*_e-to-e_ with an increase of τ as reported in Supplementary Table S1.

In the first step, calculations were performed on the isolated guest, and the isolated model dimer as a function of τ, to find the corresponding minimum potential energy *E*_*g*_ and *E*_*d-st*_, respectively. The values of the minimum potential energy relative to the model dimer, after subtraction for the corresponding absolute minimum, are reported in Supplementary Figure S1 as a function of τ (Figure 3B). It is apparent that the potential energy is almost constant for τ lower than 125°, then it gradually increases with an increase of the stretching state of the dimer.

In the second step, the most suitable relative positioning of the guest molecules with respect to the PPO model dimer to be used for initializing the minimization procedure was found through a trial-and-error process, by manually changing the relative arrangement of the host–guest pair (adduct), while treating the model dimer and the guest in low-energy conformations as rigid bodies. In practice, the guest was initially positioned with the center of mass cm_g_ (g standing for guest) pointing perpendicular to the plane of the phenyl ring in front of the corresponding center of mass cm_1_ (Figure 3B) and/or perpendicular to the end-to-end segment *d*_e-to-e_ along the direction crossing Oc (Figure 3B). The space of configurations was then sampled by casually rotating the guest around its principal axes and, for the configurations directed with cm_g_ toward Oc, also rotating the guest around the segment parallel to *d*_e-to-e_ and crossing Oc. For each trial configuration, the potential energy was calculated, and the high-energy configurations were discarded. In all cases, the lowest energies were found for configurations of the adduct in which the guest points with the center of mass toward cm_1_. Although the adopted docking scheme for finding the initial relative arrangement of the adduct is rough and not necessarily exhaustive, reliable results were found for each guest species, as shown below.

In the third and last step, the potential energy profiles were calculated starting from the identified low-energy configurations while minimizing the energy with respect to the internal coordinates of the guest, the position of its center of mass cm_g_, and the rotations around its principal axes, while fixing the value of τ. Thus, the identified minima were approached starting from different initial configurations, making our simple approach quite reliable.

**FIGURE 4:**
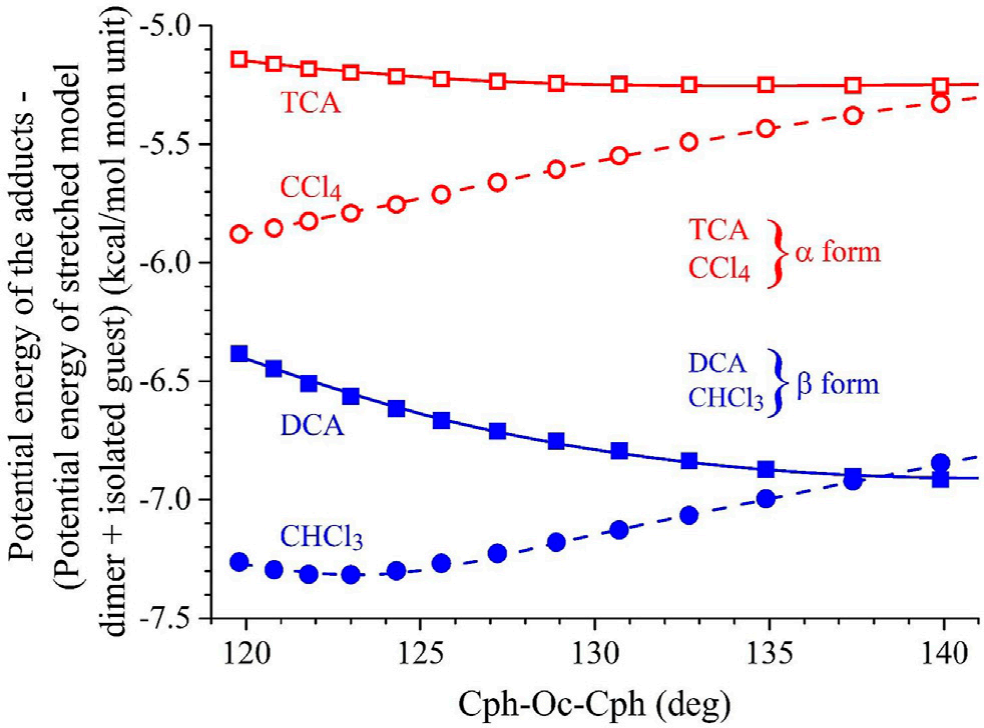
Values of the potential energy gained by the stretched model dimer of PPO in the presence of α- and β-forms guest inducer molecules (Δ*E*) as a function of the value of the valence angle at the central Oc atom Cph-Oc-Cph (τ) defined in Figure 3B. For each value of τ, the energy gain (Δ*E*) is calculated as the difference between the minimized potential energy of the adduct (guest + stretched model dimer) *E*_*a-st*_ with stretched model dimer and the sum of the potential energy of the isolated guest (*E*_*g*_) and of the isolated model dimer with τ deformed to the same value of the adduct (*E*_*d-st*_), that is, *ΔE* = *E*_*a-st*_ − (*E*_*g*_ + *E*_*d-st*_). Guest molecules such as DCA (■) and chloroform (•) are considered as examples of β-form guest inducers, whereas TCA (□) and carbon tetrachloride (○) are considered as examples of α-form guest inducers. Minima approached starting from different initial configurations that correspond, for each τ, to values of Δ*E* that are either coincident or slightly different.

In Figure 4, the potential energy gained by the model dimer of PPO in the presence of α- and β-form inducer guest molecules (Δ*E*) is shown as a function of τ. For each value of τ, the energy gain (Δ*E*) was calculated as the difference between the energy of the adduct with the stretched PPO model dimer *E*_*a-st*_ and the sum (*E*_*g*_ + *E*_*d-st*_), that is, Δ*E* = *E*_*g-st*_ − (*E*_*a*_ + *E*_*d-st*_). Therefore, the values of Δ*E* account exclusively for the dispersive energies established at the host–guest interface. The negative values of Δ*E* at a given τ value entail that the given conformation is stabilized by the guest. Notice that the lower the Δ*E*, the higher the stabilization effect.

It is apparent that by the effect of the presence of the guest molecules, a neat gain of potential energy is achieved, regardless of the τ value and guest type (Figure 4) (negative *ΔE* values). In particular, the β-form guest inducers DCA and CHCl_3_ produce a neat decrease of the potential energy by 6.5–7.5 kcal/mol, whereas the α-form guest inducers TCA and CCl_4_ produce a lower decrease of potential energy that amounts to 5–6 kcal/mol. Furthermore, whereas DCA and TCA tend to stabilize the stretched PPO conformations the higher, the greater the τ value, the potential energy stabilization tends to decrease for the ball-like molecules CCl_4_ and CHCl_3_ with increasing τ. Therefore, although kinetic aspects of the guest-induced crystallization of PPO in the α- and β-forms come certainly into play, according to Figure 4, the stabilization of β-form essentially could be the result of a neat potential energy gain of the adducts with the DCA and CHCl_3_ guest molecules, regardless of the stretching state of the model dimer.

**FIGURE 5:**
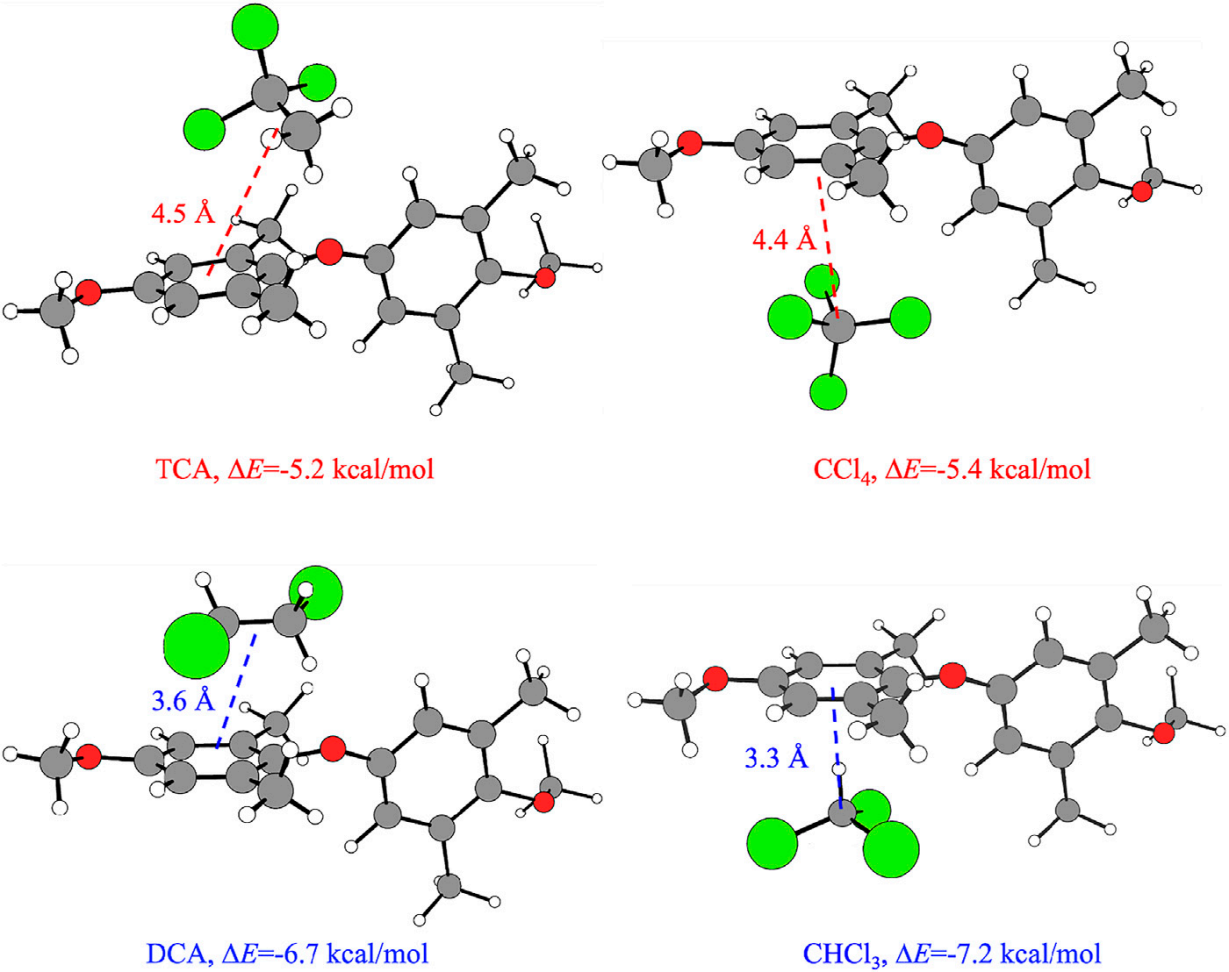
Optimized structures of the low-energy configurations of the host–guest adducts obtained from calculations, for the β- and α-forms inducers TCA and CCl_4_ (top) and DCA and CHCl_3_ (bottom), respectively. In addition, the Δ*E* values and the distance between CM_1_ of the model dimer and the approximate guest center *d*_*CM1-guest*_ are indicated for each host–guest adduct. The guest center is approximately located on the central carbon atom of the ball-like guests CCl_4_ and CHCl_3_ and the center of the C-C bond of TCA and DCA. The value of the valence angle τ is arbitrarily set equal to 130°. Similar configurations of the host–guest adducts would be obtained regardless of the value of τ. Legend: C of the guest and of the model dimer: light gray; O: red; Cl: green; H: empty spheres.

The low-energy configurations of the host–guest adducts obtained from calculations for the α- and β-form inducers are shown in Figure 5. For each host–guest adduct, the values of *ΔE* and of the distance between CM_1_ of the model dimer and the approximate guest center *d*_*CM1-guest*_ are also indicated in Figure 5. The guest center is approximately located on the central carbon atom of the ball-like guests CCl_4_ and CHCl_3_ and the center of the C-C bond of TCA and DCA. For the sake of simplicity, the model adducts of Figure 5 correspond to the configuration in which the value of the valence angle τ is arbitrarily set equal to 130°. Configurations similar to those shown in Figure 5, that is, with similar values of the *d*_*CM1-guest*_ distances and not significant differences in energy gain Δ*E*, would be obtained also for host–guest adducts including the PPO model dimer in different conformations, regardless of the value of τ (data not shown).

Inspection of Figure 5 indicates that the β-form guest inducers CHCl_3_ and DCA show a preferential interaction between a hydrogen atom and the center of the phenyl ring of the PPO dimer, while the α-forms guest inducers CCl_4_ and TCA preferentially interact *via* a chlorine atom with the center of the phenyl ring. Such a kind of interaction between a halogen atom and a phenyl ring has already been reported (Milano et al., 1998). Furthermore, the guests inducing the β-form show a shorter *d*_*CM1-guest*_ distance than the guests inducing the α-form. In particular, the values of the host–guest distances *d*_*CM1-guest*_ are 3.6 and 3.3 Å for the β-form guest inducers DCA and CHCl_3_, respectively, and 4.5 and 4.4 Å for the α-form guest inducers TCA and CCl_4_, respectively. At the same time, the shorter distances *d*_*CM1-guest*_ also reflect the tendency of the β-form inducers DCA and CHCl_3_ to establish more favorable interactions with the PPO units, as the energy gain in terms of *ΔE* is higher than the one achieved by the α-form inducers TCA and CCl_4_ (Figure 4).

**FIGURE 6:**
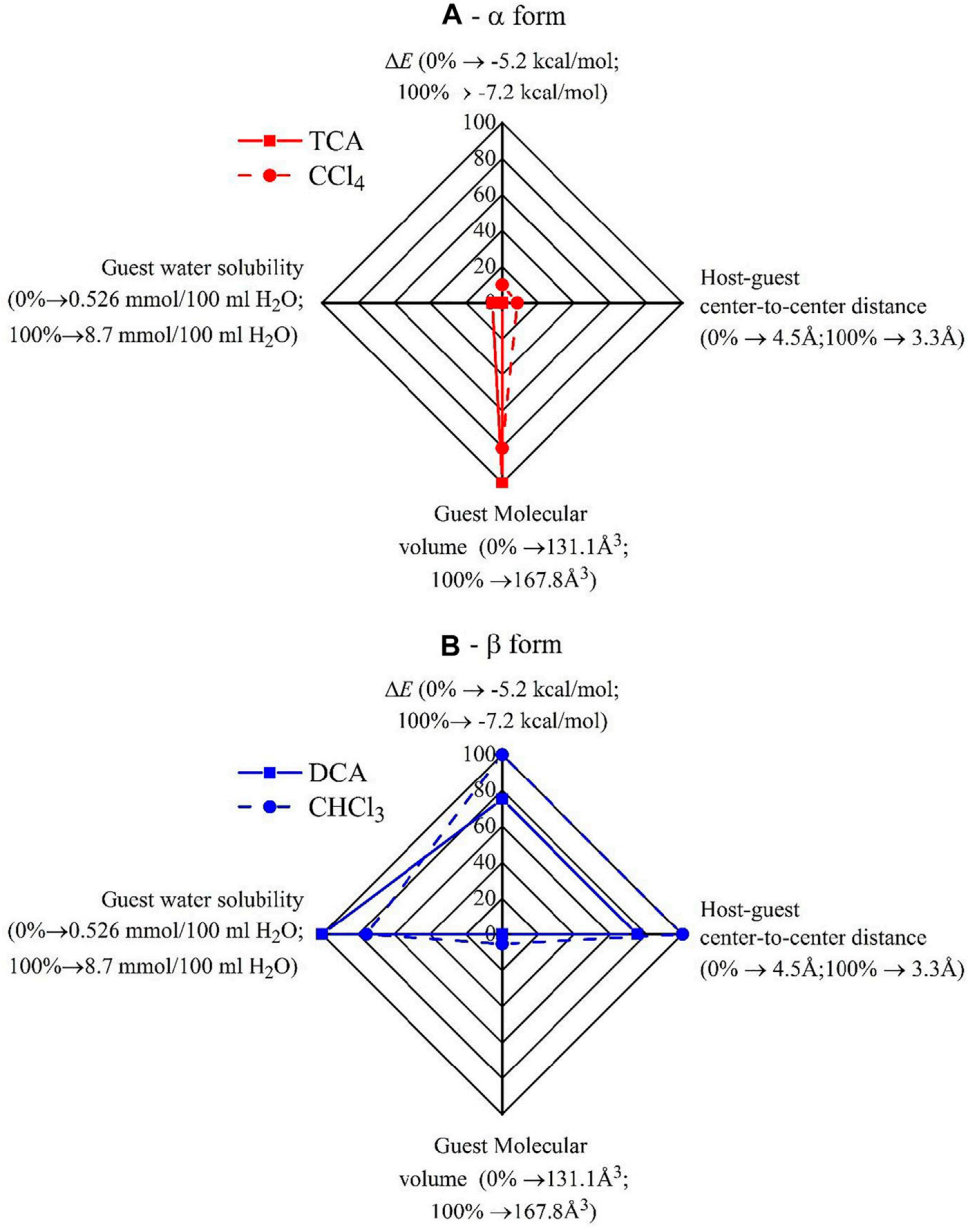
Kiviat diagrams illustrating the role of the leading parameters controlling the guest-induced crystallization of PPO in CC and NC α- **(A)** and β-forms **(B)**. The lowest and the highest values of the parameters were scaled to 100 and 0%, respectively, for Δ*E* and *d*_*CM1-guest*_, and to 0 and 100%, respectively, for the molecular volume and water solubility.

In Table 4, the values of Δ*E* and *d*_*CM1-guest*_ relative to the model adducts of Figure 5 are compared with the values of the leading parameters that have been identified in the preceding session (Tables 1–3) as the ones which control the formation of the CC and NC α- and β-forms, that is, the water solubility and the volume of the molecule. The corresponding Kiviat diagrams are reported in Figure 6. The four axes of the Kiviat diagram correspond to the potential energy gain achieved by the PPO model dimer by the effect of the presence of the guests (Δ*E*), the host-guest center-to-center distance (*d*_*CM1-guest*_), the guest molecular volume, and the water solubility of the guests. These parameters’ lowest and highest values were scaled to 100 and 0%, respectively, for Δ*E* and *d*_*CM1-guest*_, and to 0 and 100%, respectively, for the molecular volume and water solubility.

**TABLE 4:** Values of the relevant parameters that control the formation of the CC and NC α- and β-forms of PPO, deduced from calculations (Δ*E* and *d*_*CM1-guest*_) and empirical correlations (molecular volume and water solubility) of DCA and CHCl_3_ as examples of β guest inducers and TCA and CCl_4_ as an example of α guest inducers.

Guest molecules	Δ*E*—models of Figure 5 (kcal/mol)	*d*_*CM1-guest*_ (Å)	Guest molecular volume (Å^3^)	Guest solubility in 100 ml H_2_O (mmol)	Crystalline form
TCA	−5.2	4.5	167.8	0.967	α-form
CCl_4_	−5.4	4.4	161.0	0.526	α-form
DCA	−6.7	3.6	131.1	8.700	β-form
CHCl_3_	−7.2	3.3	133.1	6.701	β-form

The data of Figure 6 and Table 4 indicate that, in agreement with the data of Tables 1–3, more hydrophilic and smaller guest molecules favor the formation of the CC and NC crystalline β-form. Based on the potential energy calculations (Figures 4, 6B; Table 4), the β-form inducer guests are also those that establish highly favorable interactions with PPO units and form adducts with a low center-to-center distance (3.3–3.6 Å), at least in the case of DCA and CHCl_3_. The less hydrophilic guest molecules TCA and CCl_4_ that possess a higher molecular volume, instead, (Figure 6A) establish less favorable interactions with PPO units, form adducts with higher center-to-center distance (4.4–4.5 Å), and thus favor the crystallization of α-form.

## 4 Conclusion

This study aimed to establish guest molecular features determining the formation of α or β CC and NC phases of PPO. This aim was pursued by collecting literature data and by adding many new film preparations (both by solution casting and by guest sorption in amorphous films) to evaluate the influence of the chemical nature of the guest on the formation of α or β phases.

The present analysis shows that independently of the two considered crystallization methods, the α-form is favored by hydrophobic and bulky guest molecules while the β-form (being characterized by a higher chain periodicity) is favored by hydrophilic and small guest molecules. In detail, all the considered guests with molecular volumes higher than 230 Å^3^ and lower than 149 Å^3^ lead to the α- and β-forms, respectively. Moreover, all guests with solubility lower than 0.11 mmol per 100 ml of water and higher than 2 mmol per 100 ml of water lead to the α- and β-forms, respectively.

According to our molecular modeling study on two α-form inducer guests and on two β-form inducer guests, the latter would establish stronger dispersive interactions with the polymer chain than the α-form inducers. Therefore, the achievement of CC (and derived NC) β-forms would result from the high energy gain achieved by the adduct, and the short center-to-center distance established at host-guest interfaces.

## Data Availability

The original contributions presented in the study are included in the article/Supplementary Material, and further inquiries can be directed to the corresponding author.
