# Supplementary material for: Molecular Features Behind Formation of α or β Co-Crystalline and Nanoporous-Crystalline Phases of PPO
Source: Front Chem. 2022 Jan 25;9:809850. doi: 10.3389/fchem.2021.809850 (PMC8821151; doi:10.3389/fchem.2021.809850)
Supplement: Supplementary file 1 [file Table1.pdf]

SUPPLEMENTARY INFORMATION

Molecular Features Behind Formation of α or β Co-Crystalline and Nanoporous-Crystalline Phases of PPO

Manohar Golla[1], Antonietta Cozzolino[1], Baku Nagendra[1], Emanuele Vignola[1], Christophe Daniel[1], Paola Rizzo[1], Gaetano Guerra[*,1], Finizia Auriemma[2], Massimo Christian D'Alterio[1]

[1]Dipartimento di Chimica e Biologia and INSTM Research Unit, Università degli Studi di Salerno, Via Ponte don Melillo, 84084, Fisciano, Italy

[2]Dipartimento di Scienze chimiche – Università di Napoli "Federico II", via Cintia – Complesso Monte Sant'Angelo 21, Napoli, Italy

*** Correspondence:**
Corresponding Author
gguerra@unisa.it

Table S1. Values achieved by the end-to-end distance $d_{e\text{-}to\text{-}e}$ of the model dimer of Figure 3, as a function of the value of the valence angle at the central Oc atom Cph-Oc-Cph (τ) (Figure 3).

**Cph-Oc-Cph (τ) (deg)**	$d_{e\text{-}to\text{-}e}$ **(Å)**
119.8	9.8
120.8	9.9
121.8	10.0
123.0	10.1
124.3	10.2
125.6	10.3
127.2	10.4
128.9	10.5
130.7	10.6

132.7	10.7
134.9	10.8
137.4	10.9
139.9	11.0
142.5	11.1
145.2	11.2

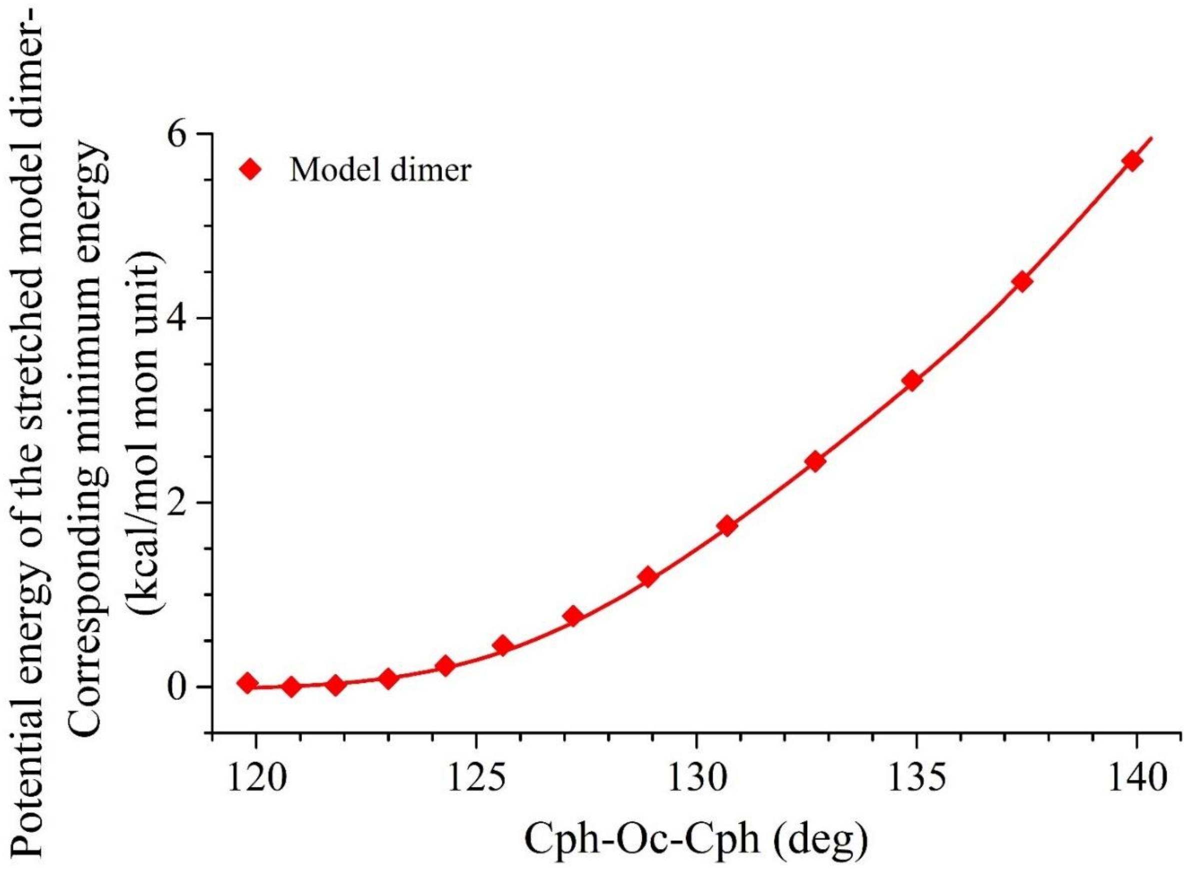

Figure S1. Minimum values of potential energy of the model dimer minus the potential energy of the corresponding absolute minimum calculated as a function of the value of the valence angle at the central Oc atom Cph-Oc-Cph (τ) (Figure 3).
